# Stemness of Normal and Cancer Cells: The Influence of Methionine Needs and SIRT1/PGC-1α/PPAR-α Players

**DOI:** 10.3390/cells11223607

**Published:** 2022-11-15

**Authors:** Youssef Siblini, Farès Namour, Abderrahim Oussalah, Jean-Louis Guéant, Céline Chéry

**Affiliations:** 1 INSERM, UMR_S1256, NGERE—Nutrition, Genetics, Environmental Risk Exposure, University of Lorraine, 54500 Vandoeuvre-lès-Nancy, France; 2 Reference Centre of Inborn Metabolism Diseases and Department of Molecular Medicine, University Hospital Center, 54500 Vandoeuvre-lès-Nancy, France

**Keywords:** normal stem cells, cancer stem cells, methionine, methionine dependence, stemness, sirtuin 1, peroxisome proliferator-activated receptor gamma coactivator 1-alpha, peroxisome proliferator-activated receptor alpha, S-adenosylmethionine

## Abstract

Stem cells are a population of undifferentiated cells with self-renewal and differentiation capacities. Normal and cancer stem cells share similar characteristics in relation to their stemness properties. One-carbon metabolism (OCM), a network of interconnected reactions, plays an important role in this dependence through its role in the endogenous synthesis of methionine and S-adenosylmethionine (SAM), the universal donor of methyl groups in eukaryotic cells. OCM genes are differentially expressed in stem cells, compared to their differentiated counterparts. Furthermore, cultivating stem cells in methionine-restricted conditions hinders their stemness capacities through decreased SAM levels with a subsequent decrease in histone methylation, notably H3K4me3, with a decrease in stem cell markers. Stem cells’ reliance on methionine is linked to several mechanisms, including high methionine flux or low endogenous methionine biosynthesis. In this review, we provide an overview of the recent discoveries concerning this metabolic dependence and we discuss the mechanisms behind them. We highlight the influence of SIRT1 on SAM synthesis and suggest a role of PGC-1α/PPAR-α in impaired stemness produced by methionine deprivation. In addition, we discuss the potential interest of methionine restriction in regenerative medicine and cancer treatment.

## 1. Introduction

Stem cells are an unspecialized population of cells that exhibit self-renewal capacity and can generate other cell types [1]. The stemness level defines the capacity of the cells to produce other differentiated cells. For instance, the stemness level of the cell varies from a totipotent stem cell that can give rise to a whole organism to a pluripotent stem cell, such as embryonic stem cells (ESCs) or their induced counterpart, induced pluripotent stem cells (iPSCs), that can give rise to all cell types of the three germ layers (ectoderm, endoderm, and mesoderm), down to a unipotent cell that only gives rise to one type of cells [1]. On the other hand, there is a cancer subpopulation that shares similar characteristics with normal stem cells, frequently called cancer stem cells (CSCs) or tumor-initiating cells (TICs) [2]. This subpopulation demonstrates upregulated pluripotency gene expression, can give rise to other cancerous cell types, and has been blamed for cancer relapse and treatment resistance [3,4,5,6]. In addition, recent discoveries shed light on a new similarity in the stemness capacity between cancer stem cells and normal stem cells. This similarity lies in a metabolic dependence on methionine (Met) [3,4,5,7,8,9,10,11,12]. Met is an essential amino acid that is fundamental for protein synthesis. The Met cycle is linked to one-carbon metabolism, which is essential for many biological processes, including methylation and nucleic/amino acid synthesis [13]. In this review, we described recent studies concerning the stemness capacity of cells and Met, highlighting the influence of sirtuin 1 (SIRT1) on SAM synthesis and suggesting the role of peroxisome proliferator-activated receptor gamma coactivator 1-alpha (PGC-1α) and peroxisome proliferator-activated receptor alpha (PPAR-α) in impaired stemness produced by methionine deprivation. We also discussed potential strategies that have taken advantage of this dependence on stem cells for the progress of regenerative medicine and to help treat recurrent cancer.

## 2. Methionine Cycle and One-Carbon Metabolism

**Figure 1:**
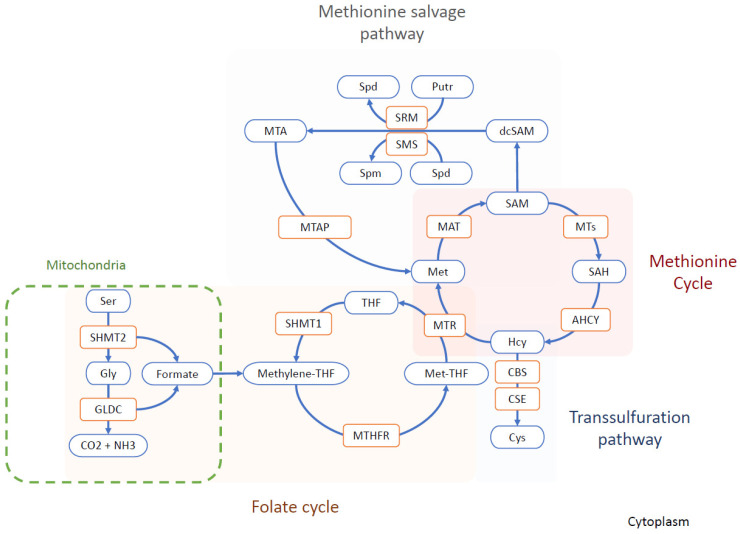
Simplified chart of the one-carbon metabolism. In the methionine cycle, methionine is converted into SAM via MAT. SAM is a universal methyl donor. SAM is converted to SAH in transmethylation reactions by methyltransferases. The resulting SAH is converted to homocysteine, which can either pass through the transsulfuration pathway, forming cystein in 2 steps catalyzed by CBS and CSE, or remethylated to methionine by MS, using the methyl group provided by Met-THF. The remethylation regenerates THF, which is then used to produce methylene-THF and Met-THF via SHMT1 and MTHFR, respectively. Methylene-THF can also be generated in the mitochondria by pathways that involve SHMT2 and GLDC. In the methionine salvage pathway, SAM decarboxylation produces an aminopropyl group donor for Putr and Spd synthesis. The donation of the aminopropyl group is catalyzed by aminopropyl transferases and produces MTA. MTA is converted back to SAM via MTAP. Abbreviations: AHCY, adenosylhomocysteinase; CBS, cystathionine beta synthase; CO2, carbon dioxide; CSE, cystathionine γ-lyase; Cys, cysteine; dcSAM, decarboxylated S-adenosylmethionine; GLDC, glycine decarboxylase; Gly, glycine; Hcy, homocysteine; MAT, methionine adenosyltransferase; methylene-THF, methylenetetrahydrofolate; Met-THF, methyltetrahydrofolate; MTA, methylthioadenosine; MTAP, methylthioadenosine phosphorylase; MTHFR, methylenetetrahydrofolate reductase; MTR, methionine synthase; MTs, methyltransferases; NH3, ammonia; Putr, putrescine; SAH, S-adenosylhomocysteine; SAM, S-adenosylmethionine; Ser, serine; SHMT1, serine hydroxymethyltransferase 1; SHMT2, serine hydroxymethyltransferase 2; SMS, spermine synthase; Spd, spermidine; Spm, spermine; SRM, spermidine synthase;; THF, tetrahydrofolate.

One-carbon metabolism is central to many biological processes that are crucial for cell development and survival. This metabolic network allows the synthesis of nucleic acid (purines and thymidine), maintains the homeostasis of the amino acid (glycine, serine, and Met), and permits epigenetic regulations [13]. The Met cycle plays a crucial role in epigenomic mechanisms. Met is converted into S-adenosyl methionine (SAM) through methionine adenosyltransferase (MAT). SAM is the universal methyl donor for methylation reactions, including histone and DNA methylation [14]. SAM is then converted into S-adenosylhomocysteine (SAH), after giving away its methyl group through histone methyltransferase (HMT) or DNA methyltransferase (DNMT) [14]. SAH is, in turn, converted to homocysteine (Hcy) that can either pass through the transsulfuration pathway or be converted back to Met by methionine synthase (MS) [14]. The Met cycle is tightly linked to the folate cycle. Met, folate, and cobalamin are required in the interconnected reactions [13]. For this reason, the one-carbon metabolism is understood as a potential link between environmental factors, such as nutrition and epigenomic mechanisms [15,16]. The Met salvage pathway illustrates the metabolic importance of Met, also called the 5′-methylthioadenosine (MTA) cycle. In this pathway, SAM is decarboxylated by adenosylmethionine decarboxylase 1 (AMD1), which is then used to donate the propylamine group to polyamines (putrescine and spermidine) by spermidine synthase (SRM) and spermine synthase (SMS). This results in the formation of spermidine and spermine, respectively, with methylthioadenosine (MTA) as a by-product. MTA is further processed by methylthioadenosine phosphorylase (MTAP) to regenerate Met [17] (Figure 1). 

The one-carbon unit production is compartmentalized between the mitochondria, cytosol, and during the S- and G2/M-phases of the cell cycle in the nucleus [13,18]. The mitochondrial one-carbon units are produced by the glycine decarboxylase (GLDC) reaction [19]. GLDC is a rate-limiting enzyme in the serine–glycine pathway that catalyzes glycine degradation into ammonium, CO_2_, and 5,10-methylenetetrahydrofolate (methylene-THF) [19]. On the same note, ALDH1L2, the mitochondrial form of 10-formyltetrahydrofolate dehydrogenase (FDH), plays an essential role in the distribution of one-carbon units between the mitochondria and cytosol via an NADP (+)-dependent reaction [20]. On the other hand, the cytosolic source of one-carbon units is obtained through the serine hydroxymethyltransferase 1 (SHMT1) rate-limiting reaction [13,19]. SHMT1 catalyzes the conversion of serine and tetrahydrofolate to glycine and 5,10-methylene tetrahydrofolate [13,19].

In the Met cycle, the concentration of SAM in the cells is highly buffered by glycine N-methyltransferase (GNMT), an enzyme that uses the methyl group from SAM to convert glycine into sarcosine (monomethylglycine), thus regulating the SAM/SAH ratio [21,22,23]. Excess Met intake is counteracted by increased MAT and GNMT reactions [24]. Although GNMT is mainly expressed in the liver, there is some evidence of its expression in stem cells [25].

## 3. Methionine and Stemness

Several one-carbon metabolism genes, such as MAT2A, GLDC, SHMT1, and ALDH1L2, have been shown to demonstrate differential expression in stem cells compared to their differentiated counterparts [3,4,12,26]. In addition, Met restriction or inhibition of the upregulated genes showed a close link with the stemness capacities of cells, paving the way to novel strategies by exploiting this metabolic dependence in stem cells [4,5,8,9,10,12].

Several studies have shown the importance of Met in the maintenance of the self-renewal capacities and stemness of normal and cancerous stem cells. [3,4,5,8,9,10,12]. We review the recent studies that show the link between Met and the stemness of normal and cancer stem cells.

### 3.1. Methionine in Normal Stem Cells

The work of Shiraki et al. on human embryonic stem cells (hESCs) and human induced pluripotent stem cells (hiPSCs) highlighted the high-Met metabolic state of these cells compared to differentiated cells and their dependence on Met in a concentration-dependent manner. Their work was inspired by the specialized metabolic state of stem cells and the dependence of mouse ESCs on threonine catabolism [27]. Mouse embryonic stem cells demonstrate a high-flux metabolic state with a high expression of threonine dehydrogenase (TDH). The TDH enzyme catalyses a rate-limiting step in the mitochondrial conversion of threonine into glycine and acetyl-coenzyme A (CoA), which are essential for the folate cycle and tricarboxylic acid (TCA) cycle, respectively. This makes mouse ESCs critically dependent on threonine for their survival, pluripotency maintenance, and differentiation [27]. Given that threonine dehydrogenase is a non-functional pseudogene in humans, Shiraki et al. sought an equivalent approach to detect whether a similar effect could be observed with other amino acids in human cells by depriving them of single amino acids and measuring the total number of cells 48 h after deprivation. The most striking results were observed in the Met-deprived condition [12]. Furthermore, they showed that short Met deprivation led to a fast decrease in intracellular SAM with a consequent decrease in H3K4me3, and a decrease in NANOG expression, along with an increase in the differentiation potency in the three germ layers [12]. It is also interesting to note that the supplementation of Met increased NANOG expression and decreased the proportion of p53+ cells in a concentration-dependent manner [12]. In contrast, the prolonged Met deprivation effect on the cells was irreversible, with G0/G1 phase arrest triggering apoptosis. This leads to the conclusion that hESCs/hiPSCs do not rely on threonine-derived one-carbon metabolism for self-renewal and pluripotency, and alternatively use Met metabolism to achieve the same result [11,12]. Recently, the same team showed the involvement of zinc signaling in the regulation of stem cell pluripotency and differentiation. Culturing PSCs in Zn-deprived medium partially mimicked methionine deprivation (e.g., potentiated differentiation), showing altered methionine metabolism-related metabolite profiles. Likewise, the depletion of methionine reduced protein-bound Zn, which includes MS, through displacement with homocysteine [28].

The increase in Met requirements in the stemness and self-renewal capacity of ESCs/iPSCs is related to SAM levels and the synthesis of SAM by MAT2A and MAT2B. Knocking down MAT2A or MAT2B hampers the cell’s ability to transform Met into SAM and leads to a decreased self-renewal capacity that can be rescued with SAM addition. The Met salvage pathway seems to play a limited role in the adaption of ESCs/iPSCs cells to their increased Met needs. The knocking down of SMS, the enzyme synthesizing spermine, impairs the Met salvage pathway, but does not phenocopy the Met depletion condition [12]. In addition, cycloleucine, an analog of Met that specifically inhibits MAT, decreases SAM levels and cell growth without affecting Met or SAH levels. Met restriction induces the upregulation of MAT2A expression as a way for the cells to possibly increase SAM levels and cope with the restrictive conditions [12]. These results indicate that SAM, rather than Met itself, is essential for the self-renewal and survival of stem cells. However, in cells with knocked down SIRT1, MAT2A is downregulated, and the upregulation induced through Met restriction leads to a limited increase in MAT2A level, compared to the wild-type (WT) condition. This downregulation is triggered by c-Myc, a proliferation proto-oncogene transregulator under the control of SIRT1 through a deacetylation mechanism that increases its stability and activity [10].

**Figure 2:**
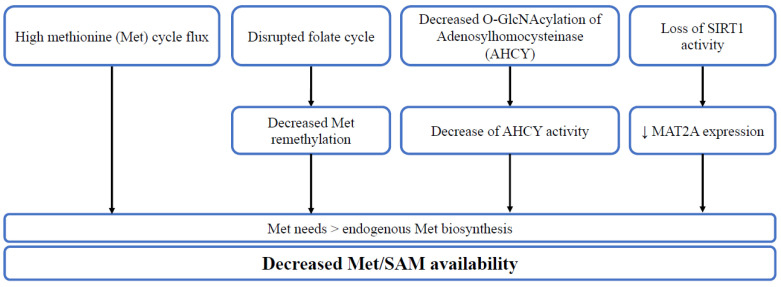
The diverse mechanisms leading to decreased Met/SAM availability. High methionine cycle flux, disrupted folate cycle, decreased O-GlcNAcylation of adenosylhomocysteinase (AHCY) or loss of SIRT1 activity lead to higher methionine needs in comparison to endogenous synthesis, thus decreasing methionine/SAM availability.

Adenosyl homocysteinase (AHCY) is another target enzyme of the Met cycle, which may influence the relation between SAM and cell stemness. AHCY catalyzes the hydrolysis of S-adenosylhomocysteine (SAH) to generate adenine and homocysteine as a part of the Met cycle [8]. Inducing the differentiation of mESCs decreases the mRNA levels of AHCY, MAT2A, MAT2B SAM and SAH, and homocysteine metabolites of the Met cycle. Depleting AHCY using shRNA leads to a decrease in the SAM/SAH ratio and pluripotency markers, notably Oct4 and Nanog, and an increase in the differentiation markers. Furthermore, the depletion results in an increase in cell number in the G1 phase, accompanied by a reduction in cells in the S phase and activation of the p53-dependent signaling pathway, leading to increased apoptosis [8]. The decrease in the SAM/SAH ratio leads to a decrease in H3K4me3 levels, notably at the Pou5f1 and Nanog loci and the O-GlcNAc post-translational modifications on serine or threonine residues of nucleocytoplasmic proteins [8]. O-GlcNAcylation is a nutrient-responsive modification with a pivotal role in stem cell biology [29,30,31]. This post-translational modification modulates enzyme activities. AHCY undergoes T136 O-GlcNAcylation, which promotes its activity by increasing its tetrameric assembly and its affinity with Hcy [8]. Inducing mESC differentiation leads to a gradual decrease in AHCY O-GlcNAcylation with reduced enzyme activity, thus regulating mESC pluripotency and self-renewal capacity [8] (Figure 2).

### 3.2. Methionine in Cancer Stem Cells

The growth of many cancer cells depends on Met cellular availability [32,33,34]. The Met dependency of cancer cells is defined by the inability of cells to proliferate in a medium deprived of Met, even when the metabolic precursor of Met, Hcy, is present [32,33,34]. Recent studies have shown that cancer stem cells are Met-dependent. The disruption of one-carbon metabolism enzymes or the reduction in Met hamper the self-renewal capacity and pluripotency of cancer stem cells [3,4,5,7,9].

Zgheib et al. found evidence of Met dependency in glioblastoma cancer stem cells, but not in the related adherent differentiated cells. The cells’ tumorsphere formation capacities are recovered upon adding folate or MeTHF major molecules in the folate cycle. Glioblastoma stem cells demonstrated a disrupted folate cycle and could not furnish the necessary one-carbon unit to convert it into Met and SAM [3]. In contrast, the Met dependency stems from the high need for SAM in lung TICs [4]. Lung TICs have a high Met cycle flux, leading to their dependency on exogenous Met, as observed for normal stem cells. Compared to their differentiated counterpart, these cells demonstrate high GLDC expression and activity [4]. The high activity of GLDC in TICs redirects the flux of one-carbon units towards the increased demand of the Met cycle [4,35]. Knocking down this gene decreases Met and SAH levels to a level equivalent to differentiated cells, decreases histone methylation, and hampers the tumor-initiating capacities of these cells, further demonstrating the importance of Met [4]. Interestingly, recent studies have shown the importance of GLDC in maintaining and inducing pluripotency in ESCs and iPSCs through fueling H3K4me3 modification, while knocking down GLDC suppressed the pluripotency state [26,36,37]. Transient (48 h) Met starvation of lung TICs led to more striking results, with a decrease in SAM, SAH, and histone methylation levels and again hampered tumor-initiating capacities [4]. Supplementing cells with Hcy does not allow the cells to recover from the effect of Met starvation, in agreement with the Met dependency definition described above. Adding SAM or replating cells in Met-supplemented media allowed the cells to recover and regain their tumor-initiating ability [4]. Moreover, three other enzymes, MAT2A, MTHFR, and SHMT2, are also elevated in TICs compared to differentiated cells, further confirming the impact of GLDC upregulation of TICs on Met cycle flux. Furthermore, in cases where any of these genes are knocked down, the cell’s ability to form tumors is hampered, similarly knocking down GLDC [4]. Both MAT2A and MTHFR were found to be upregulated in lung tumors, but only MAT2A was found to be upregulated in high-grade primary tumors or metastasis, highlighting the possibility of using MAT2A inhibitors, such as FIDAS-5, to treat patients with aggressive lung tumors. Wang et al. found that it hampers the tumor-initiating ability of TICs [4]. The mechanisms of Met dependency observed in TICs from glioblastoma and lung cancer cells is, thus, due to the disruption of the balance between folate and methionine cycles, leading to excess or impaired production of methyl-THF and methionine, which limits their growth [34]. Overall, these results suggest a prominent role of one-carbon metabolism in conferring stemness in cancer cells [3,34,38].

The dependence on Met in glioblastoma TICs seems to have a bell shape depending on the concentration used, with an optimal concentration. Therefore, lower or higher concentrations lead to lower tumorsphere formation capacity [3]. Such a correlation was not investigated in normal stem cells, as the limit of the tested concentration of Met was approximately 120 µM in comparison to an upper concentration of 500 µM in the study on glioblastoma TICs [3,7,12]. In normal stem cells, the concentration of Met was positively correlated with the increase in pluripotency gene expression and lower mortality rates were observed with a higher concentration of Met [12]. Cultivating breast cancer stem cells in Met-deprived conditions mirrors the results obtained in normal stem cells cultured in similar conditions [5,9]. The mammosphere formation assay shows the significantly lower capacity of cancer stem cells to form in those conditions, possibly through the same mechanism observed in ESCs/iPSCs with a recovered capacity upon adding SAM [5,9]. In addition, the Met-deprived condition leads to the increase in MAT2A levels as a homeostatic response and a decrease in H3K4me3 levels with lower levels of SOX9 markers and CD44+/CD24−. Intriguingly, these conditions potentiate the knocking down effect of MAT2A or its inhibition by MAT2A inhibitors. Similarly, mice fed a Met-restricted diet and treated with MAT2A inhibitor show a potentiated result, with increased apoptosis, lower tumor volume, and metastatic capacity compared to the control conditions [5]. Breast cancer stem cells were also found to highly express the O-GlcNAc transferase enzyme (OGT) and O-GlcNAcylation [9]. In line with what was found in normal stem cells, altering OGT and O-GlcNAc levels hampers stem cells’ ability to form mammospheres in vitro, tumor formation in vivo, and lowered the levels of CD44+CD24− [9]. In addition, OGT seems to be a regulator of epithelial–mesenchymal transition (EMT) and cancer stem-like cell markers, including CD44, NANOG, and c-Myc [9]. Likewise, O-GlcNAcylation is involved in regulating stem cell marker expression in colon cancer cells [39]. Despite the resemblance in the importance of the O-GlcNAc effect on the stemness between normal and cancer stem cells, they are not linked to AHCY. Thus, the influence of O-GlcNAc on the Met cycle in cancer stem cells needs to be assessed in future studies [9].

### 3.3. The Influence of SIRT1 and PPAR-Alpha/PGC1-Alpha Pathway

SIRT1 and PPAR-α are key players in the links between Met metabolism and cell stemness. SIRT1 is one of the seven mammalian proteins that belong to the sirtuin family [14,40,41]. It catalyzes histone and non-histone lysine deacetylation in a NAD+-dependent manner [14,40,41]. Previous work in our lab showed the role of SIRT1 in regulating energy metabolism through PPAR-α and PGC-1α in a methyl-deficient diet [14,40,41,42,43]. PGC-1α is a master regulator of lipid metabolism and fatty acid oxidation. It is regulated by methylation and acetylation. The deacetylation of the PGC-1α protein leads to its activation and is known to coactivate PPAR-α to enhance the expression of fatty acid oxidation genes, antioxidant enzymes, and mitochondrial biogenesis [40]. Methyl-deficient diets decrease the expression of SIRT1 and subsequent activation of PGC-1α through imbalanced acetylation and methylation of the latter dysregulating energy metabolism [14,40,41]. The impaired expression and/or activity of methionine synthase in fibroblasts from patients with mutations in MTR and/or other inherited disorders of vitamin B12 metabolism also result in decreased protein expression of SIRT1, which plays a key role in the underlying pathological mechanisms of these disorders [40]. 

Sirtuin 1 (SIRT1) is involved in iPSC formation [44]. SIRT1 knockdown decreased, while resveratrol (RSV) increased the efficiency of iPSCs. SIRT1 enhances iPSC generation through deacetylation of p53, inhibition of p21 and enhancement of Nanog expression [45]. SIRT1 has been demonstrated to interact and acetylate Oct4 to maintain the stemness of naive pluripotent stem cells [46]. Sirt1 also deacetylates Sox2 through direct interaction with Oct4 [47]. The B12 and folate deficiency decreases the endogenous synthesis of methionine and decreases the brain expression of miR-34a in pups from deficient mother rats [48]. Of note, miR-34a reduces the reprogramming efficiency through inhibition of SIRT1 expression [45]. Decreased SIRT1 leads to the stabilization and increased activity of the P53 protein through its increased acetylation at the K120 and K164 sites [49]. This SIRT1-dependent upregulation of P53 activity is effective in undifferentiated hESCs, but not in other cell types. SIRT1 plays a role in DNA damage repair that is crucial for hESCs’ fast mitotic division, which is prone to replication-related DNA errors [50]. It leads to programmed cell death through decreased expression of DNA repair enzymes, such as MSH2, MSH6 and APEX1, in hESCs [50]. Furthermore, SIRT1 is essential for telomere elongation during the iPSC generation process [51]. The level of expression of SIRT1 has been demonstrated to be elevated in normal and cancer stem cells (CSC), compared to their differentiated counterparts [45,46,50,51,52,53,54]. However, there is a debate on the role of SIRT1 in CSCs with its double functionality as a tumor suppressor and promoter [55,56]. SIRT 1 expression is increased, and its activity is critical for stemness and cell survival in cancer stem cells from glioma, colon and liver cancer, and leukemia [57]. SIRT1 inhibits DNMT3A and promotes the expression of SOX2 through promoter-reduced methylation [54]. It also increases the expression of other stemness-associated genes, including Oct4, Nanog, Cripto, Tert and Lin28, in colon cancer stem cells [58]. 

Several studies have investigated the influence of Met restriction on the effects of SIRT1 on pluripotency. However, whether SIRT1 influences stemness through its decreased expression produced by the impaired remethylation pathway of Met metabolism is unknown. Kilberg et al. showed that SIRT1 knock-out produces effects similar to those caused by Met restriction observed by Shiraki et al., in mouse embryonic stem cells (mESCs). The SIRT1 knock-out (KO) mESCs specifically impaired Met metabolism [10]. The metabolomic analysis of the SIRT1 KO cells cultured in complete media showed an elevated Met level, along with a decrease in SAM levels. The restriction of Met led to elevated differentiation markers and induced apoptosis with high sensitivity to Met deprivation, compared to other amino acids [10]. Interestingly, culturing SIRT1 KO mESCs in a complete medium with normal levels of Met demonstrated SAM levels similar to those of WT mESCs after Met restriction, suggesting that knocking out SIRT1 is somewhat equivalent to Met restriction, even with normal Met levels in culture medium [10]. Furthermore, SIRT1 KO reduced NANOG and OCT4 expression, with a marked decrease in NANOG expression when cultured in Met-restricted medium. In line with these results, the relative levels of H3K4me3 at the transcription starting site of the Nanog gene were reduced by both SIRT1 deletion and Met restriction, possibly through the same mechanism observed in hESCs/hiPSCs and steered by SAM levels [10]. 

PPAR-α is a key player in stemness. It triggers the expression of key genes of pluripotency reprogramming and enables pluripotent cells to adapt to their metabolic needs [59,60,61,62]. A Food and Drug Administration (FDA)-approved PPAR-α agonist was found to facilitate iPSC generation and enhance their programming efficiency by increasing the expression of pluripotency genes, including Nanog, Nr5A2, Oct4, and Rex1 [60]. PPAR-α knockdown of human glioma stem cells by shRNA reduces in vitro proliferation and inhibits orthotopic xenograft tumor growth [59]. Furthermore, PPAR-alpha was shown to play an important role in promoting mammosphere formation by modulating the expression of stem cell genes, including Jagged1, via the NF-κB/IL6 axis [62]. The PPARα-specific agonist treatment increases the number of mammospheres [62]. On the other hand, PPARα siRNA conditions decrease the number of mammospheres [62]. Whether the decreased activity of PPAR-α is related to the inactivation of PGC-1α by SIRT1 in stem cells has not been considered in experimental studies. However, this hypothesis is consistent with the role of SIRT1 activity in stem cells and its relationship with the cellular synthesis of SAM. 

**Figure 3:**
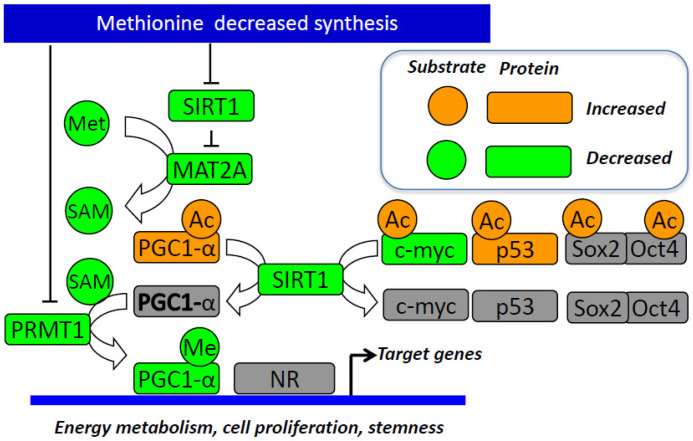
Summary of the links between decreased methionine endogenous synthesis and stemness. The decrease in methionine synthesis reduces SAM availability and SIRT1 and PRMT1 expression, which lead to decreased methylation and increased acetylation of PGC-1α and the subsequent impaired coactivation of nuclear receptors and other target genes of energy metabolism and pluripotency. The decreased SIRT1 reduces MAT2A expression, thus contributing to the decrease in the SAM levels. The decreased SIRT1 activity also leads to increased acetylation and reduced stability of p53 and increased acetylation of c-Myc and interacting Sox2 and Oct4, which are the three key genes of pluripotency reprograming. These mechanisms suggest that methionine endogenous synthesis/availability influence stemness capacity through its effects on the expression on key actors of pluripotency reprogramming and energy metabolic needs of stem cells. Abbreviations: Ac, acetyl group; c-Myc, MYC proto-oncogene; MAT2A, methionine adenosyltransferase 2A; Me, methyl group; Met, methionine; NR, nuclear receptor; PGC-1α, peroxisome proliferator-activated receptor gamma coactivator 1 alpha; PRMT1, protein arginine methyltransferase 1; SAM, S-adenosylmethionine; SIRT1, sirtuin 1.

Given the link between SIRT1, PGC-1α and PPAR-α and their relation to stemness, we speculate that depriving cells of methionine would decrease SIRT1 levels and lead to the subsequent dysregulation of the energy metabolism through PGC-1α/PPAR-α [42], thus, participating in the hampered stemness induced by the decreased SAM levels. The data found in the literature point out the interplay between methionine restriction, SIRT1 and the PGC-1α/PPAR-α axis in stemness reprogramming and the need for further studies to produce a more integrated and mechanistic view of this interplay (Figure 3).

## 4. Targeting Methionine Diet and/or Metabolism in Therapeutic Strategies

The metabolic dependence of stem cells on Met paved the way for new strategies that can be used in regenerative medicine and novel therapeutic approaches for cancer treatment. For instance, targeting Met dependency in iPSCs can eliminate leftover iPSCs after cardiac differentiation in transplanted cells to prevent tumor formation upon engraftment [63,64]. Cultivating engineered cardiac tissue in Met-free culture conditions at 42 °C led to the decreased expression of Lin28, OCT3/4, and NANOG without negatively impacting the tissue. Cardiac tissue showed spontaneous and synchronous beating, while maintaining or upregulating the expression of various cardiac and extracellular matrix genes [63,64]. Furthermore, a recently established protocol used the deprivation of both methionine and zinc to generate functional endocrine β cells [28].

A Met-restricted diet or treatment with an MAT2A inhibitor is a potential way to treat cancer by the targeted elimination of the cancer stem cell population [4,5,6,34]. Several of the previously mentioned studies have tested the impact of using Met restriction/deprivation or MAT2A inhibitors on the cancer stem cell population. Met restriction could be a metabolic primer for cancer cell death in combination with other strategies [65]. The targetable vulnerabilities have been studied thoroughly in preclinical studies with an expanding list of targets [66]. For example, Met restriction enhances the chemotherapeutic sensitivity of colorectal cancer stem cells by the miR-320d/c-Myc axis [67]. In addition, a recent clinical study showed that dietary Met restriction for patients with adjuvant transarterial chemoembolization (TACE) might be beneficial, as the pro-stemness capacities may be attributed to the activation of the Met cycle [68]. In addition, L-methioninase, an enzyme that catalyzes the degradation of l-Met to methanethiol, α-ketobutyrate, and ammonia, could be an additional therapeutic approach [69]. Recombinant methioninase inhibits the self-renewal and proliferation of gastric cancer stem cells. In vivo experiments demonstrated that HA-coated nanoparticles that co-encapsulated plasmid methioninase and 5-Fu enhance the targeting ability and promote the inhibition effects on tumor growth in gastric cancer [70]. These therapeutic approaches raise the question of their influence on normal stem cell pools in the human body, although normal stem cells have been shown to partially utilize Hcy to recover a part of the Met pool [12]. Future studies are needed to better understand Met dependency as a common hallmark of normal and cancer stem cells and help to build personalized approaches for cancer treatment [4,5,71].

## 5. Conclusions

Met dependence is a common feature of normal and cancer stem cells produced by diverse mechanisms specific to certain cell lines, including the influence of decreased synthesis of endogenous Met, and increased flux of the Met cycle. Convergent evidence shows that SIRT1 and PPAR-α/PGC-1α could be involved in the cause and/or consequences of Met dependency and stemness capacities (Figure 2 and Figure 3). Cultivating stem cells in Met-restricted/deprived conditions alters their stemness capacity by halting SAM generation, which decreases histone methylation levels, notably H3K4me3, and alters stemness gene expression (Table 1 and Table 2). This suggests that targeting the Met dependence mechanism could be useful in regenerative medicine and recurrent cancer treatment.

## Figures and Tables

**Table 1:** Highlights of recent publications in relation to methionine restriction outcome and SIRT1/PGC-1α/PPAR-α players in the stemness of normal stem cells/embryos.

Normal Stem Cells				
Cell Type	Experimental Condition	Observed Changes		Reference
		Increased	Decreased	
hESCs/iPSCs	Short Met deprivation	Differentiation potency Salvage pathway MAT2A expression p53-p38 signaling	SAM H3K4me3 mark NANOG Homocysteine	[12]
	Prolonged Met deprivation	Apoptosis (if not exposed to differentiation signals) G1-G0 arrest		
mESCs	SIRT1 KO or KD	Differentiation Sensitivity to methionine restriction (mimics methionine restriction)	MAT2A SAM H3K4me3 + other histone marks NANOG and OCT4	[10]
	SIRT1 KO or KD + Met restriction	Differentiation Apoptosis	MAT2A expression SAM levels H3K4me3 + other histone marks NANOG expression	
Mice embryos	SIRT1 KO embryos	Sensitivity to maternal methionine restriction	Mat2a expression SAM levels H3K4me3 mark	
	SIRT1 KO embryos + Met restriction	Developmental defects	Growth (retardation) Survival rate	
mESCs	Depletion of AHCY	Differentiation p53-dependent signaling pathway Apoptosis	SAM H3K4me3 NANOG and OCT4	[8]
	Blocking O-GlcNAcylation of AHCY	Teratomas formation in vivo Differentiation	AHCY activity SAM levels H3K4me3 mark NANOG and OCT4 expression	
iPSCs	SIRT1 knockdown		iPSC formation (during the initiation phase of reprogramming)	[45]
	Resveratrol (SIRT1 activation)	iPSC formation (acts on the initiation phase of reprogramming)		
iPSCs	Sirt1 KO	Chromosome/chromatid breaks	Telomeres length after several cycles	[51]
mESCs	Sirt1 KO	Acetylation of Oct4 Fgf5 and Otx2 expression Maintenance of Oct4 expression Primed pluripotency network	Nanog and Klf2	[46]
hESCs	SIRT1 inhibition	DNA damage p53 activation Cell death	DNA repair enzyme levels (such as MSH2, MSH6, and APEX1)	[50]
iPSC	PPARα agonist	Nanog expression (reprogramming-promoting effect)		[60]

**Table 2:** Highlights of recent publications in relation to methionine restriction outcome and SIRT1/PGC-1α/PPAR-α players in the stemness of cancer stem cells.

Cancer Stem Cells				
Cell Type	Experimental Condition	Observed Changes		Reference
		Increased	Decreased	
Triple-negative breast CSCs	Met restriction	MAT2A Sensitivity to MAT2A inhibition	Mammosphere formation CD44(hi)/C24(low) CSC population Sox9 expression H3K4me3 mark	[5]
Lung CSCs	Met restriction		SAM levels H3K4me3 and other histone marks Colony-forming abilities in vitro Tumorigenic potential in vivo Cell-surface expression of CD166	[4]
Glioblastoma CSCs	Standard limiting dilution of Met	Mitochondrial SHMT2 and ALDH1L2 SOX2, OCT4, NANOG	Cytoplasmic SHMT1, MTHFD1 and DHFR	[3]
Breast CSCs	Inhibition of OGT (potential relation to methionine cycle)		Mammosphere formation CD44(hi)/C24(low) CSC population NANOG+ population ALDH+ population c-Myc+ population	[9]
Colorectal carcinoma CSCs	SIRT1 knockdown/inhibition		Stemness-associated genes (including Oct4, Nanog, Cripto, Tert and Lin28) Abilities of colony and sphere formation Percentage of CD133+ cells Tumorigenicity in vivo	[58]
Liver CSCs	SIRT1 knockdown/inhibition		Cell growth of liver CSCs Sphere and clone formation efficiencies in vitro Tumorigenic potential in vivo SOX2, Nanog and Oct4 expression levels	[54]
	Overexpression of exogenous SIRT1	Self-renewal of liver non-CSCs Clone and sphere formation efficiencies Tumorigenic potential in vivo		
Glioma CSCs	PPARα KD	Astrocytic differentiation	Tumorigenicity of in vivo Proliferative capacity and clonogenic potential in vitro Tumorigenicity of orthotopic xenografts Stem cell markers (SOX2, c-Myc and nestin)	[59]
Liver CSCs	SIRT1 inhibition	Susceptibility to chemotherapeutic drugs Senescence via activation of p53-p21 and p16 pathway	Stemness-associated genes (including NANOG, SOX2, OCT4, CD13, CD44 and EpCAM) Spheroid formation Tumorigenicity in vivo	[52]
Breast CSCs	PPARα agonist	Mammosphere formation NF-κB/IL6 axis Mammosphere regulatory genes		[62]

## Data Availability

Not applicable.

## Abbreviations

AHCY, adenosylhomocysteine; AMD1, adenosylmethionine decarboxylase 1; CoA, coenzyme A; CSCs, cancer stem cells; DNMT, DNA methyltransferase; EMT, epithelial-mesenchymal transition; ESCs, embryonic stem cells; FDA, Food and Drug Administration; FDH, 10-formyltetrahydrofolate dehydrogenase; GLDC, glycine decarboxylase; GNMT, glycine N-methyltransferase; Hcy, homocysteine; hESCs, human embryonic stem cells; hiPSCs, human induced pluripotent stem cells; HMT, histone methyltransferase; iPSCs, induced pluripotent stem cells; KO, knock-out; MAT, methionine adenosyltransferase; mESCs, mouse embryonic stem cells; Met, methionine; methylene-THF, 5,10-methylenetetrahydrofolate; MS, methionine synthase; MTA, 5′-methylthioadenosine; MTAP, methylthioadenosine phosphorylase; OGT, O-GlcNAc transferase; PGC-1α, peroxisome proliferator-activated receptor gamma coactivator 1 alpha; PPAR-α, peroxisome proliferator-activated receptor alpha; SAH, S-adenosylhomocysteine; SAM, S-adenosylmethionine; SHMT1, serine hydroxymethyltransferase 1; SIRT1, sirtuin 1; SMS, spermine synthase; SRM, spermidine synthase; TACE, transarterial chemoembolization; TCA, tricarboxylic acid; TDH, threonine dehydrogenase; TICs, tumor-initiating cells; WT, wild type.
